# Authentication of Edible Bird’s Nest Beverage-Derived Powders Using Block-Based Near-Infrared Hyperspectral Imaging and Multi-Model Agreement Mapping

**DOI:** 10.3390/foods15203676

**Published:** 2026-10-16

**Authors:** Krairuek Ngowsuwan, Sunee Jungtheerapanich, Anupun Terdwongworakul

**Affiliations:** 1 Nondestructive Quality Evaluation Commodities Technology Laboratory, Kasetsart Agricultural and Agro-Industrial Product Improvement Institute (KAPI), Kasetsart University, Bangkok 10900, Thailand; aapsnj@ku.ac.th; 2 Department of Agricultural Engineering, Faculty of Engineering at Kamphaeng Saen, Kasetsart University, Nakhon Pathom 73140, Thailand; fengant@ku.ac.th

**Keywords:** edible bird’s nest, near-infrared hyperspectral imaging, food authentication, multiple-instance learning, random forest, PLS-DA, spatial mapping

## Abstract

Authentication of edible bird’s nest (EBN) in heterogeneous matrices is challenging because whole-region spectral averaging may obscure localized variation. This study developed a block-based near-infrared hyperspectral imaging (NIR-HSI) framework integrating complementary classifiers and spatial multi-model agreement mapping. The development dataset comprised 90 independently prepared physical samples, each with four independently sampled and repacked scans; external validation used 21 additional physical samples (84 scans). Spectra (900–1700 nm) were represented by mean-spectrum or block-based approaches and classified using partial least squares discriminant analysis (PLS-DA), random forest (RF), and Multiple Instance Adaptive Cosine Estimator (MI-ACE). Model development used leave-one-physical-sample-out cross-validation. In external validation, mean-spectrum PLS-DA achieved accuracies of 0.7143–0.8571, whereas mean-spectrum RF correctly classified all 21 samples. For 9 × 9 blocks, PLS-DA achieved accuracies of 1.0000, 0.9048, and 1.0000 across the three preprocessing conditions, while RF and MI-ACE correctly classified all 21 samples under all conditions. All-ROI analysis identified a significant second-derivative difference at approximately 1505.65 nm between EBN-positive and EBN-free classes (U = 0, *p* < 0.001). This region is potentially informative but should not be considered EBN-specific. Overall, the framework provided proof-of-concept evidence of strong physical-sample-level classification while preserving spatial information.

## 1. Introduction

Edible bird’s nest (EBN), produced primarily from the salivary secretions of *Aerodramus* swiftlets, is a high-value animal-derived food product composed mainly of proteins and carbohydrates, along with minerals, lipids, and other minor constituents [1]. Its high commercial value and limited availability make EBN and EBN-containing products particularly susceptible to economically motivated adulteration with lower-cost substitutes, such as karaya gum, *Tremella* fungus, agar, and egg white [1,2]. Reliable authentication methods are therefore essential for ensuring product integrity and supporting quality assurance throughout the EBN supply chain [2,3,4,5].

A range of analytical techniques has been investigated for EBN authentication, including microscopy, electrophoresis, deoxyribonucleic acid (DNA)-based methods, chromatography, vibrational spectroscopy, proteomics, and mass spectrometry [2,3]. Targeted chemical approaches, including those based on peptide and oligosaccharide markers [3,4], can provide high analytical specificity but generally require dedicated instrumentation, sample preparation, and skilled personnel. In contrast, spectroscopic techniques enable rapid acquisition of chemical fingerprint information with minimal reagent use, making them attractive for sample screening [5,6]. When coupled with chemometric modeling, spectroscopy has also been successfully applied to authenticity assessment in other high-value food commodities, demonstrating the broader utility of spectral fingerprints for detecting differences associated with authenticity and adulteration [7].

Near-infrared hyperspectral imaging (NIR-HSI) combines spectral and spatial information by acquiring a spectrum at each spatial location within an image [8,9,10,11]. This capability has supported a wide range of applications in food quality assessment, contaminant detection, process monitoring, and authentication [8,9,10,11,12]. NIR-HSI has also been applied to EBN for quantitative mapping of protein, carbohydrate, and sialic acid when appropriate chemical reference measurements were available [13]. Recent image-based learning approaches have also demonstrated the growing use of deep feature extraction, transfer learning, and attention-based architectures for agricultural-product quality inspection [14,15]. However, authentication of processed EBN-derived matrices presents a different analytical challenge because EBN may be present at low concentrations within heterogeneous formulations, while direct chemical reference information may not be available for individual spatial locations.

The representation of hyperspectral data is therefore a critical consideration in model development. Mean-spectrum analysis reduces dimensionality and local spectral variability by averaging spectra across the entire region of interest (ROI), but this process inevitably removes spatial information. In contrast, pixel-wise analysis preserves maximum spatial detail but may be more susceptible to noise, scattering effects, and surface irregularities [12,16,17]. Spatially averaged blocks provide an intermediate representation that can reduce pixel-level variability while retaining localized spectral information that would otherwise be lost through whole-ROI averaging [12,16,17,18].

Recent advances in hyperspectral-image classification, particularly in remote-sensing applications, have increasingly focused on sophisticated spatial–spectral representation learning through few-shot learning, state-space/Mamba architectures, graph-based modeling, attention mechanisms, and global–local feature integration [19,20,21,22,23]. Although these approaches demonstrate the potential of advanced representation learning for extracting complex spatial–spectral patterns, their effective application generally depends on sufficient independent training data and reliable labels. In the current EBN authentication setting, the number of independent physical samples was comparatively modest, and direct chemical labels were unavailable at the individual-block level. A simpler block-based representation was therefore adopted to preserve localized spectral information while maintaining a transparent relationship between the measured spectra and the resulting classification models.

Classifier selection may further influence the performance achieved with a given spectral representation. Partial least squares discriminant analysis (PLS-DA) is widely used for high-dimensional, collinear spectroscopic data, whereas random forest (RF) provides a nonlinear ensemble-learning approach capable of capturing complex interactions among predictors [24,25,26,27]. However, the absence of direct reference labels for individual spatial blocks presents an additional challenge for conventional supervised classification. This limitation motivates the use of multiple-instance learning, in which class labels are available at the scan or bag level but not necessarily at the individual-instance level. Multiple-instance learning has been applied to weakly labeled image-classification problems in which informative local instances must be inferred from coarse bag-level labels [28]. In this context, the Multiple Instance Adaptive Cosine Estimator (MI-ACE) provides a framework for target characterization under imprecise instance-level labeling [29]. Because PLS-DA, RF, and MI-ACE rely on distinct learning principles, examining the agreement among their localized predictions may provide complementary information for spatial interpretation [30].

Accordingly, this study developed an integrated block-based NIR-HSI framework for authenticating EBN beverage-derived powders while preserving localized spectral information. The framework integrates supervised classifiers (PLS-DA and RF), weakly supervised MI-ACE to address the absence of direct block-level reference labels, and spatial multi-model agreement mapping to visualize localized concordance and disagreement among classifier predictions. Mean-spectrum PLS-DA and RF were included as whole-ROI reference approaches to assess whether retaining localized spectral information provided additional value over conventional spectral averaging. Model development and internal evaluation were conducted using leave-one-physical-sample-out cross-validation (LOPSO-CV), in which all four scans originating from the same physical sample were withheld together to preserve independence at the physical-sample level. Generalization was subsequently assessed using an independent external-validation set comprising additional physical samples not used during model development. Finally, the selected block-based classifiers were integrated into the spatial agreement-mapping framework to characterize localized variation in classification decisions within individual samples.

## 2. Materials and Methods

### 2.1. Sample Preparation

EBN material was obtained directly from a commercial swiftlet-house farm in Prachuap Khiri Khan Province, Thailand. The same EBN material was used for preparation of the EBN-containing formulations and for the 100% ground EBN spectral reference. The beverage formulations were independently prepared by a contract manufacturer following specifications provided by the researchers. Each physical sample was produced as an independent 500 g batch. The development dataset comprised 90 physical samples, including 45 EBN-containing samples and 45 EBN-free controls. The EBN-containing samples were prepared at five EBN levels: 0.1, 0.5, 1.0, 2.0, and 3.0% (*w*/*w*), with nine independently prepared physical samples at each level. The EBN-free controls consisted of samples prepared using karaya gum (KA; *n* = 20) or sodium alginate (SO; *n* = 25) as the principal structural material in place of EBN. Sodium alginate was included as an EBN-free matrix control because it was also present as a major structural component in the EBN-containing formulations, allowing evaluation of whether the classifiers could distinguish EBN-associated spectral information from the alginate-containing matrix.

The formulations of the laboratory-prepared samples are summarized in Table 1. For the EBN-containing formulations, the amount of EBN was varied while the combined mass of EBN and sodium alginate was maintained at 200 g per 500 g batch. Each formulation additionally contained 30.00 g of rock sugar, 0.10 g of gellan gum, 0.03 g of sucralose, and 269.87 g of water. The SO control contained 200 g of sodium alginate without EBN, whereas the KA control contained 200 g of karaya gum without EBN or sodium alginate.

All beverage samples were converted into dried powders prior to NIR-HSI analysis. Each sample was first filtered to separate the insoluble fraction from the liquid phase. The retained solids were washed with 50 mL of distilled water per washing cycle for six cycles to reduce residual sugars and other water-soluble constituents. The washed solids were allowed to drain on a sieve and were subsequently dried in a hot-air oven at 70 °C for 18 h. When residual moisture was visually evident, drying was extended as required for no more than an additional 2 h, giving a maximum total drying time of 20 h. The dried material was ground using a mortar and pestle and passed through a 60-mesh sieve to obtain a uniform powder suitable for hyperspectral imaging. This preparation procedure was intended to reduce strong spectral contributions from water and readily soluble formulation components while retaining the solid fraction for subsequent EBN authentication. Because the analytical workflow involved filtration, repeated washing, oven drying, grinding, and sieving, the complete procedure should not be regarded as a direct or nondestructive analysis of the original beverage. Rather, the present method was designed for laboratory classification of prepared beverage-derived powder fractions prior to NIR-HSI measurement. The drying step represented the major elapsed-time requirement, requiring approximately 18–20 h. Filtration, six washing cycles, grinding, and sieving additionally required repeated manual handling, although the hands-on processing time for these individual operations was not separately recorded. Accordingly, the sample-preparation procedure was considered an integral part of the analytical workflow rather than a preliminary step independent of the proposed authentication procedure.

An independent external-validation set comprising 21 additional physical samples was used to assess model generalization. This set consisted of nine laboratory-prepared EBN-containing samples at 0.1, 0.5, and 1.0% (*w*/*w*), with three physical samples at each level; six commercial ready-to-drink products with label-declared EBN content obtained from three commercial brands, with two samples per brand; and six EBN-free controls consisting of three KA and three SO samples. According to the product labels, the declared EBN contents were 1.1%, 2.8%, and 1.1% for Brands 1, 2, and 3, respectively, expressed on the basis of dry EBN weight before cooking. The commercial products were assigned to the EBN-positive class based solely on their product-label declarations; no independent chemical confirmation or quantification of EBN content was performed. Accordingly, their class assignments should be interpreted as label-based reference classifications rather than analytically confirmed EBN-positive ground truth. All external-validation samples were processed into dried powders using the same filtration, washing, drying, grinding, and sieving procedure described above.

In addition, one sample of 100% ground EBN powder, prepared by directly grinding authentic EBN material obtained from the same source described above, was used only as a qualitative spectral reference for subsequent comparison of spectral shapes and characteristic spectral regions. It was not used as a block-level chemical ground truth or as a reference class for model development.

### 2.2. NIR-HSI Image Acquisition and Spectral Extraction

Near-infrared hyperspectral images were acquired using a push-broom imaging system (VLNIR-CL-100-N17E, SPECIM, Oulu, Finland) covering the spectral range of approximately 900–1700 nm with a spectral resolution of approximately 3.2 nm. For each scan, approximately 2.0 g of dried powder was freshly sampled and transferred into a truncated-cone sample holder equipped with a quartz window. The diameter of the light-receiving surface was 25 mm, the rear diameter was 29 mm, and the sample cavity height was 4 mm. A lid was used to standardize sample thickness and packing conditions during image acquisition.

The sample holder was positioned approximately 3 cm from the imaging system. Images were acquired with an exposure time of 4 ms, a frame rate of 70 Hz, a scanning speed of 10 mm s^−1^, and a spatial scanning step of approximately 1 mm. Raw hyperspectral images were corrected using dark- and white-reference measurements and subsequently converted to absorbance to obtain a three-dimensional absorbance data cube with spatial dimensions X and Y and spectral dimension λ. To capture variability associated with subsampling, particle distribution, and powder packing, each physical sample was measured in four independently prepared scans. For each scan, a fresh aliquot was sampled from the dried powder and independently repacked into the sample holder before image acquisition. Thus, the four scans obtained from the same physical sample represented independently sampled and repacked aliquots rather than repeated imaging of an unchanged powder surface. Although these scans shared the same parent physical sample, differences in local particle distribution and packing could result in distinct spectral observations among scans. Consequently, the development dataset comprised 360 scan observations (90 physical samples × 4 scans), whereas the external-validation dataset comprised 84 scan observations (21 physical samples × 4 scans). Because individual pixel spectra were relatively susceptible to local noise and small-scale surface variation, spatial averaging was evaluated using 7 × 7, 9 × 9, and 11 × 11-pixel blocks. Blocks were extracted sequentially without overlap; thus, the spatial stride was equal to the corresponding block dimension in each direction. For each block, the spectra of all pixels within the block were averaged wavelength-by-wavelength to obtain one representative block spectrum. A binary sample mask was generated using the spectral image at 1218.82 nm, where sufficient contrast between the powder sample and the background was observed. Pixels corresponding to the sample were assigned a value of 1 and background pixels a value of 0. Morphological erosion equivalent to one block was subsequently applied to reduce potential edge effects and define valid regions for spectral extraction. The spatial scanning step in the scan direction was approximately 1 mm. Therefore, the 7-, 9-, and 11-pixel block lengths corresponded approximately to 7, 9, and 11 mm, respectively, along the scan direction. However, the cross-track spatial dimension was not calibrated to physical units in the present study; consequently, a complete two-dimensional physical block size could not be reliably reported and block dimensions are therefore expressed primarily in pixels.

Following spatial averaging, spectra corresponding to valid blocks within the ROI were extracted. Occasional not-a-number (NaN) or infinite (Inf) values present in the extracted spectra, originating from absorbance conversion in spectral regions with low signal-to-noise ratio (SNR), were replaced by linear interpolation along the wavelength axis using adjacent valid spectral values. The corrected block spectra constituted the block-based spectral representation, with each valid block retained as an individual spectrum together with its original spatial coordinates.

**Figure 1:**
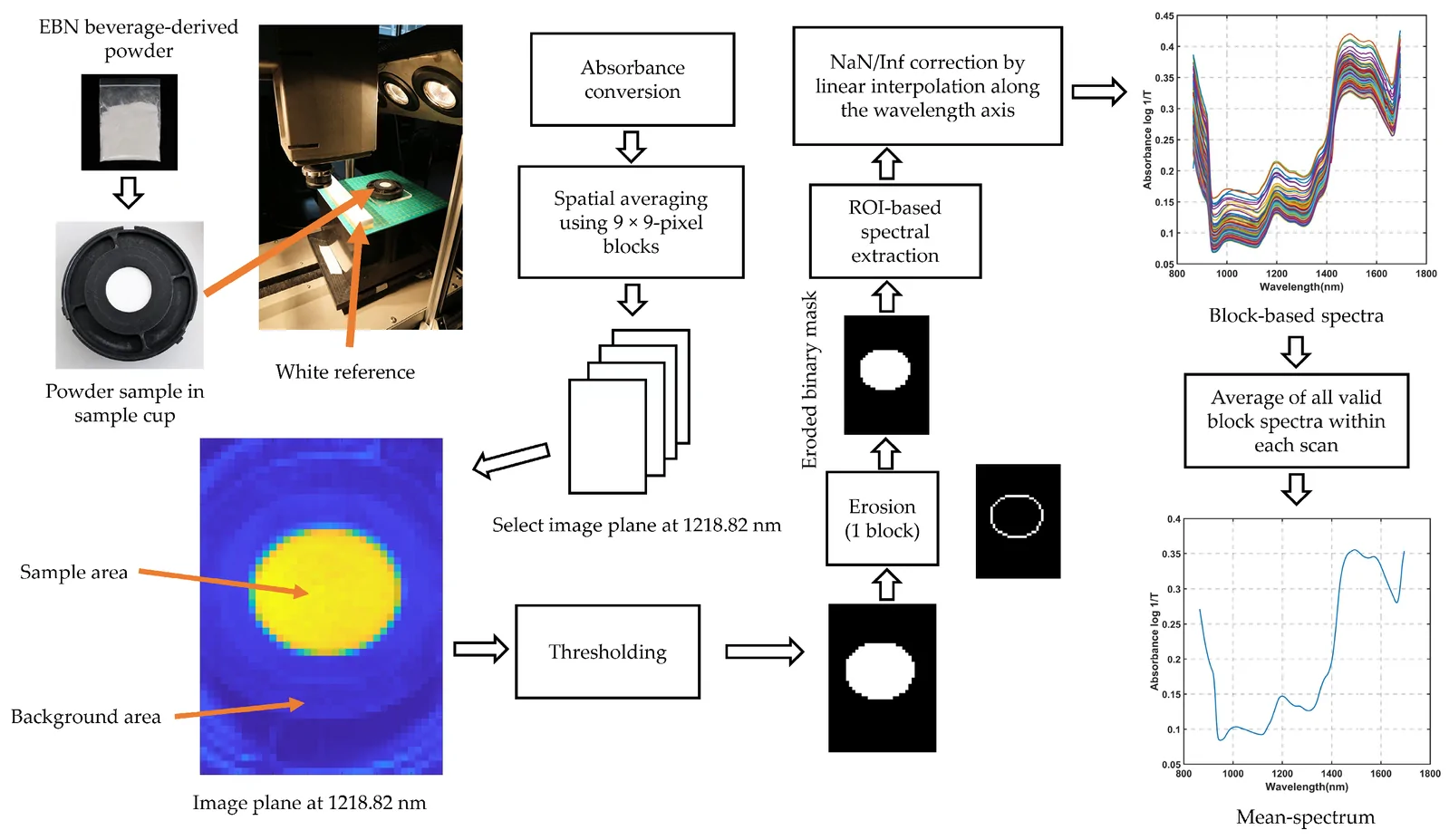
Workflow of NIR-HSI image processing and extraction of block-based and mean-spectrum spectral representations. Arrow and line colors are used for visual clarity only and do not indicate different classes or categories.

The mean-spectrum representation was subsequently generated from the same corrected block-based spectra by averaging all valid block spectra within each scan, yielding one representative spectrum per scan. This resulted in 360 scan-level mean spectra nested within 90 independent physical samples in the development dataset (four scans per physical sample) and 84 scan-level mean spectra nested within 21 independent physical samples in the external-validation dataset. Thus, the scan-level spectra were treated as repeated observations nested within their parent physical samples rather than as independent experimental units. The block-based representation retained localized spectral variability and spatial information, whereas the mean-spectrum representation provided a single averaged spectral profile for each scan. The overall workflow for data processing and spectral extraction is presented in Figure 1.

### 2.3. Spectral Preprocessing, Classification, and Spatial Model-Agreement Analysis

All spectral processing and classification analyses were performed in MATLAB R2024a (MathWorks, Natick, MA, USA). Three spectral conditions were evaluated for both the mean-spectrum and block-based representations: (i) absorbance spectra without additional spectral preprocessing (original), (ii) standard normal variate (SNV)-transformed spectra, and (iii) second-derivative spectra calculated using a Savitzky–Golay filter with a 21-point window and a second-order polynomial. SNV was used to reduce additive and multiplicative variations associated with light scattering and differences in particle arrangement and packing, whereas second-derivative preprocessing was used to enhance subtle spectral features and reduce baseline effects.

#### 2.3.1. Mean-Spectrum and Block-Based Classification

Two spectral data representations were evaluated: mean-spectrum and block-based spectra. EBN-containing samples were assigned to the positive class (class 1), whereas the EBN-free karaya gum (KA) and sodium alginate (SO) controls were assigned to the negative class (class 0).

For the mean-spectrum representation, PLS-DA and RF were evaluated as scan-level classifiers. Each scan was represented by a single spectrum obtained by averaging all valid block spectra within the ROI, and each model consequently generated one prediction score per scan. PLS-DA served as the linear chemometric classifier, while RF provided a nonlinear alternative for comparison.

For block-based RF, a maximum of 2000 training blocks per class was used within each cross-validation fold to reduce computational burden. Because the number of valid 9 × 9 blocks was relatively uniform across scans and physical samples, no additional scan- or sample-balanced weighting was applied for PLS-DA, and no additional sample-balanced weighting was applied for RF beyond the class-level training-block cap. For the selected 9 × 9 representation, each physical sample contributed 1074.76 ± 10.85 valid blocks across its four scans (range: 1049–1100), indicating limited imbalance in block contribution among physical samples.

MI-ACE was applied using a multiple-instance-learning formulation in which each independently repacked scan was treated as one bag and each valid block within the scan was treated as one instance. This formulation was used because the EBN-positive or EBN-negative status was known at the scan level, whereas direct chemical reference information for individual blocks was unavailable. Because direct chemical reference labels were unavailable at the individual-block level, the inherited labels used for block-based PLS-DA and RF represented weak sample-level labels rather than confirmed block-level ground truth. Blocks originating from the same scan were also correlated observations and were therefore not regarded as independent experimental units for performance assessment.

For scan-level evaluation of the block-based models, all valid blocks within the ROI of each scan were independently scored by the corresponding classifier. The continuous prediction or decision scores of the blocks belonging to the same scan were then averaged to obtain a single scan-level mean prediction score:

(1) $$\bar{s_{j}}=\frac{1}{N_{j}}\sum_{i=1}^{N_{j}}s_{i,j}$$

where $s_{i,j}$ is the prediction score of block i in scan j, $N_{j}$ is the total number of valid blocks within scan j, and $\bar{s_{j}}$ is the resulting scan-level mean prediction score. The four scan-level scores obtained from the same physical sample were subsequently averaged to obtain a single physical-sample-level score. The block-level prediction scores were retained for subsequent spatial mapping.

For PLS-DA and RF, a decision threshold of 0.5 was applied to the resulting physical-sample-level score; scores equal to or greater than 0.5 were classified as EBN-positive, whereas scores below 0.5 were classified as EBN-negative.

For MI-ACE, the raw adaptive cosine estimator (ACE) decision score was treated as a spectral similarity measure rather than a calibrated class probability; therefore, a fixed threshold of 0.5 was not imposed. Instead, a model-specific decision threshold, $T_{MI-ACE}$, was determined from the development data during model training and cross-validation, as described in Section 2.3.2. Physical-sample-level scores equal to or greater than the model-specific threshold were classified as EBN-positive, whereas scores below this threshold were classified as EBN-negative. The threshold obtained for the final MI-ACE model was subsequently fixed and applied unchanged to the independent external-validation set.

For the mean-spectrum PLS-DA and RF models, a single continuous prediction score was generated for each scan. The prediction scores from the four independently sampled and repacked scans originating from the same physical sample were subsequently averaged to obtain one physical-sample-level score before class assignment using a threshold of 0.5, as described in Section 2.3.2. Classification performance was assessed at the physical-sample level using accuracy, sensitivity, specificity, balanced accuracy, the area under the receiver operating characteristic curve (AUC), precision, and F1-score. Confusion-matrix results were additionally summarized using true-positive (TP), true-negative (TN), false-positive (FP), and false-negative (FN) counts for the independent external-validation set.

#### 2.3.2. Internal Cross-Validation and Independent External Validation

Model development, hyperparameter selection, and internal performance assessment were conducted using LOPSO-CV. In each fold, all four independently sampled and repacked scans from one physical sample, together with all associated blocks, were withheld for validation, while the remaining physical samples were used for model training. The four scan-level prediction scores of the held-out sample were subsequently averaged to obtain one physical-sample-level prediction. Accordingly, internal cross-validation performance was assessed using 90 independent physical samples.

For PLS-DA, the number of latent variables (LVs) was selected by cross-validation based on residual validation variance. The minimum validation variance was used as a reference, and a more parsimonious model was selected when a lower number of LVs produced a validation variance close to the minimum and further increases in model complexity provided only marginal improvement. For RF, hyperparameters were optimized using LOPSO-CV over combinations of 50 or 100 trees, minimum leaf sizes of 1 or 5, and $\sqrt{p}$ or $\sqrt[{2}]{p}$ predictors considered at each split, where p is the number of spectral variables. For block-based RF, a maximum of 2000 training blocks per class was used within each cross-validation fold to reduce computational burden. No additional sample-balanced weighting was applied during model fitting. For the selected 9 × 9 representation, however, the number of valid blocks contributed by individual physical samples was relatively uniform, with 1074.76 ± 10.85 blocks per physical sample (range: 1049–1100), indicating limited imbalance in block contribution among physical samples. The optimal RF configuration was selected primarily according to physical-sample-level balanced accuracy, with AUC used as a secondary criterion. After hyperparameter selection, the final RF model was fitted using all available development data with the selected parameters.

For MI-ACE, covariance regularization (10^−8^, 10^−6^, 10^−4^, 10^−2^) and the maximum number of training blocks per scan (25, 50, or 100) were optimized by LOPSO-CV. Candidate configurations were ranked primarily by physical-sample-level balanced accuracy and secondarily by AUC. Within each fold, the MI-ACE decision threshold was determined from the training scans only by maximizing balanced accuracy. The final model-specific thresholds for the raw ACE decision score were 0.2308, 0.2189, and 0.2474 for the original, SNV, and second-derivative models, respectively. These thresholds were determined using the development data and subsequently fixed and applied unchanged to the independent external-validation set.

Independent external validation was performed using 21 additional independent physical samples that were not included in model development. All preprocessing procedures, selected model parameters, and decision thresholds established from the development dataset were applied without further optimization. For each physical sample, the continuous prediction scores from its four independently sampled and repacked scans were averaged to obtain a single physical-sample-level score before class assignment. External-validation performance was therefore calculated at the physical-sample level using 21 independent samples and served as the primary assessment of generalization to unseen physical samples.

Exact 95% binomial confidence intervals based on the Clopper–Pearson method were calculated for accuracy, sensitivity, specificity, and precision. For balanced accuracy, AUC, and F1-score, 95% confidence intervals were estimated using 10,000 stratified bootstrap resamples at the physical-sample level, with positive and negative samples resampled separately within their respective classes [31].

#### 2.3.3. Multi-Model Agreement Mapping

Based on the classification results, the models used for spatial multi-model agreement mapping were selected exclusively from the development dataset using LOPSO-CV, before evaluation of the independent external-validation set. For each classifier family, balanced accuracy was used as the primary selection criterion and AUC as the secondary criterion; when performance was equivalent, the least transformed spectral representation was retained as a parsimonious tie-breaking criterion. This procedure resulted in the selection of the 9 × 9 block-based second-derivative PLS-DA, SNV-preprocessed RF, and original-spectrum MI-ACE models. The independent external-validation dataset was not used for preprocessing selection, model selection, hyperparameter optimization, or threshold determination. The block-level predictions of these three fixed models were subsequently combined to evaluate spatial agreement among classifiers.

For spatial mapping, each block was independently classified by the three selected models. PLS-DA and RF block-level scores were converted to binary predictions using a threshold of 0.5, whereas MI-ACE block-level decisions were obtained using the model-specific threshold established during model development. For each block b, the binary predictions of the three models were summed as:

(2) $$V_{b}=z_{PLSDA,b}+z_{RF,b}+z_{MIACE,b}$$

where $z_{PLSDA,b}$, $z_{RF,b}$, and $z_{MIACE,b}$ represent the binary classification outcomes for block b, with 1 indicating EBN-positive and 0 indicating EBN-negative. The resulting model-agreement score $V_{b}$ ranged from 0 to 3.

The block-level scores were mapped back to their original spatial positions within the ROI. A score of 3/3 indicated that all three models classified the block as EBN-positive and was displayed in red; 2/3 was displayed in orange; 1/3 was displayed in yellow; and 0/3, indicating that all three models classified the block as EBN-negative, was displayed in white. Regions outside the ROI were displayed in black.

Because no direct chemical reference measurements were available at the individual-block level, the agreement score was not interpreted as the local concentration of EBN. Instead, it represented the degree of agreement among the three classifiers regarding EBN-associated spectral characteristics. Blocks with a score of 3/3 therefore represented consensus-positive regions, whereas blocks with a score of 0/3 represented consensus-negative regions.

Because the three classifiers were derived from the same underlying hyperspectral measurements, the model-agreement score was not interpreted as a calibrated prediction confidence or as independent evidence from separate analytical measurements. Model diversity was additionally quantified using pairwise block-level agreement, disagreement, and Cohen’s kappa for PLS-DA versus RF, PLS-DA versus MI-ACE, and RF versus MI-ACE. For each model pair, disagreement was defined as the proportion of valid blocks receiving different binary predictions from the two classifiers. These block-level statistics were used descriptively to characterize similarity and diversity among model decisions and were not treated as independent-sample performance estimates.

For subsequent spectral interpretation, spectra from 3/3 consensus-positive blocks were extracted from EBN-containing samples, whereas spectra from 0/3 consensus-negative blocks were extracted from the EBN-free KA and SO controls. The selected block spectra were averaged within each sample group and compared with the qualitative spectrum of 100% ground EBN powder to examine spectral features potentially associated with the classification of EBN-containing and EBN-free samples.

To assess whether the spectral tendency around 1505–1509 nm persisted independently of model-consensus-based region selection, an additional analysis was performed using all valid ROI blocks from the external-validation samples, irrespective of classifier prediction or agreement score. Spectra were averaged at the physical-sample level before group-level visualization. The second-derivative response at approximately 1505.65 nm was compared between EBN-positive and EBN-free physical samples using an exact two-sided Mann–Whitney U test, with the physical sample treated as the independent experimental unit.

## 3. Results and Discussion

### 3.1. Spectral Characteristics of Mean-Spectrum and Block-Based Data

The NIR-HSI data acquired from the development samples were represented using two spectral formats: mean-spectrum and block-based spectra. For the mean-spectrum representation, all valid block spectra within the ROI of each scan were averaged to obtain a single representative spectrum. This resulted in 360 mean spectra, one representing each scan. In contrast, the block-based representation retained each valid block as an individual spectrum together with its original spatial coordinates, thereby preserving localized spectral variability within each scan.

**Figure 2:**
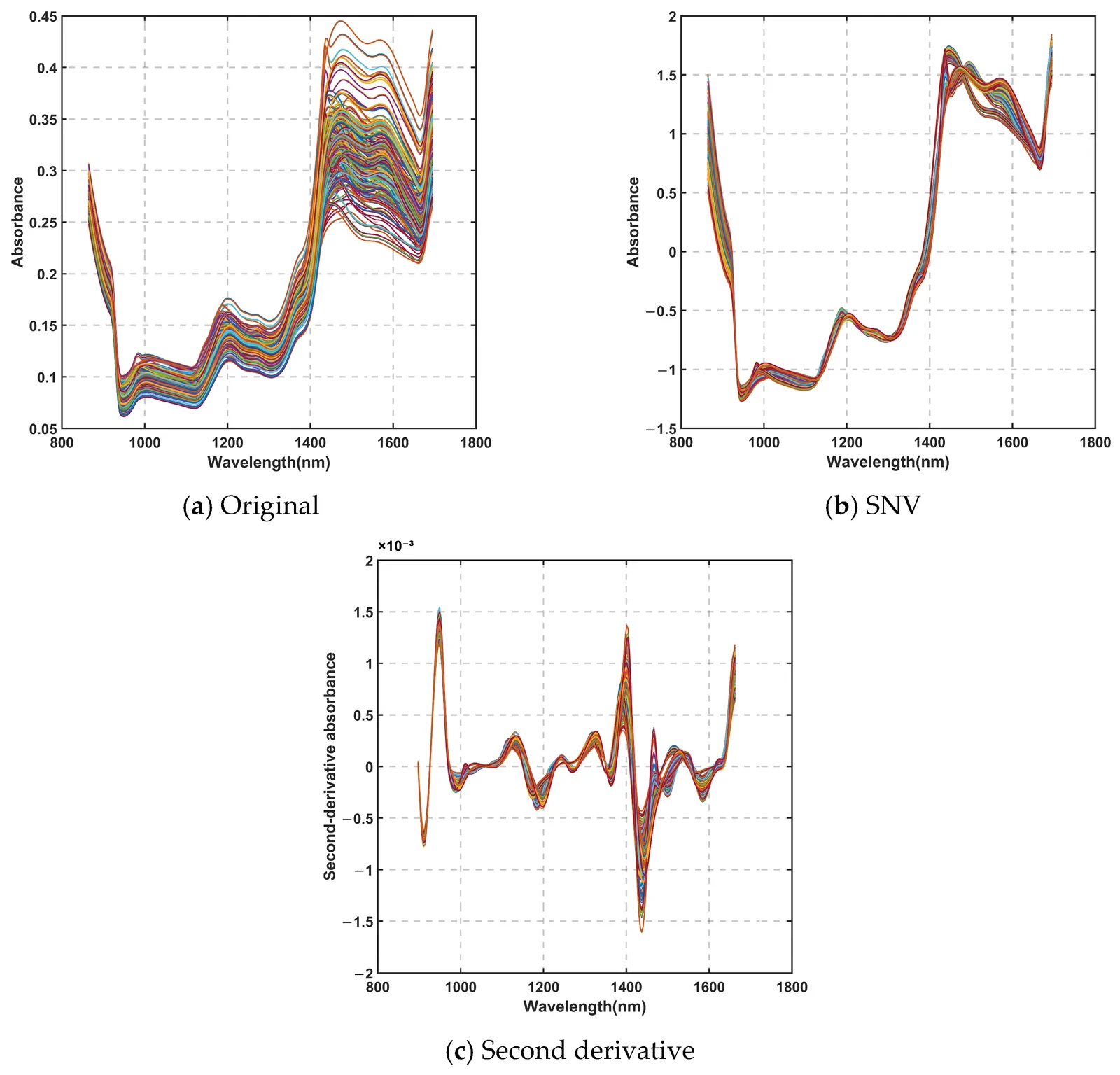
Mean-spectrum near-infrared spectra obtained from the development samples: (**a**) Original spectra, (**b**) SNV preprocessed spectra, and (**c**) Second-derivative spectra. Line colors are used only to visually distinguish overlapping spectra and do not indicate different sample classes or categories.

The mean-spectrum data under the three spectral conditions—original, SNV, and second derivative—are shown in Figure 2. The original spectra exhibited differences in spectral intensity and baseline, which may be associated with variations in particle distribution, packing density, surface characteristics, and light scattering among independently sampled and repacked aliquots. SNV preprocessing reduced much of the additive and multiplicative variation, resulting in improved alignment of the spectral profiles. Second-derivative preprocessing further reduced baseline effects and enhanced subtle differences in spectral shape.

**Figure 3:**
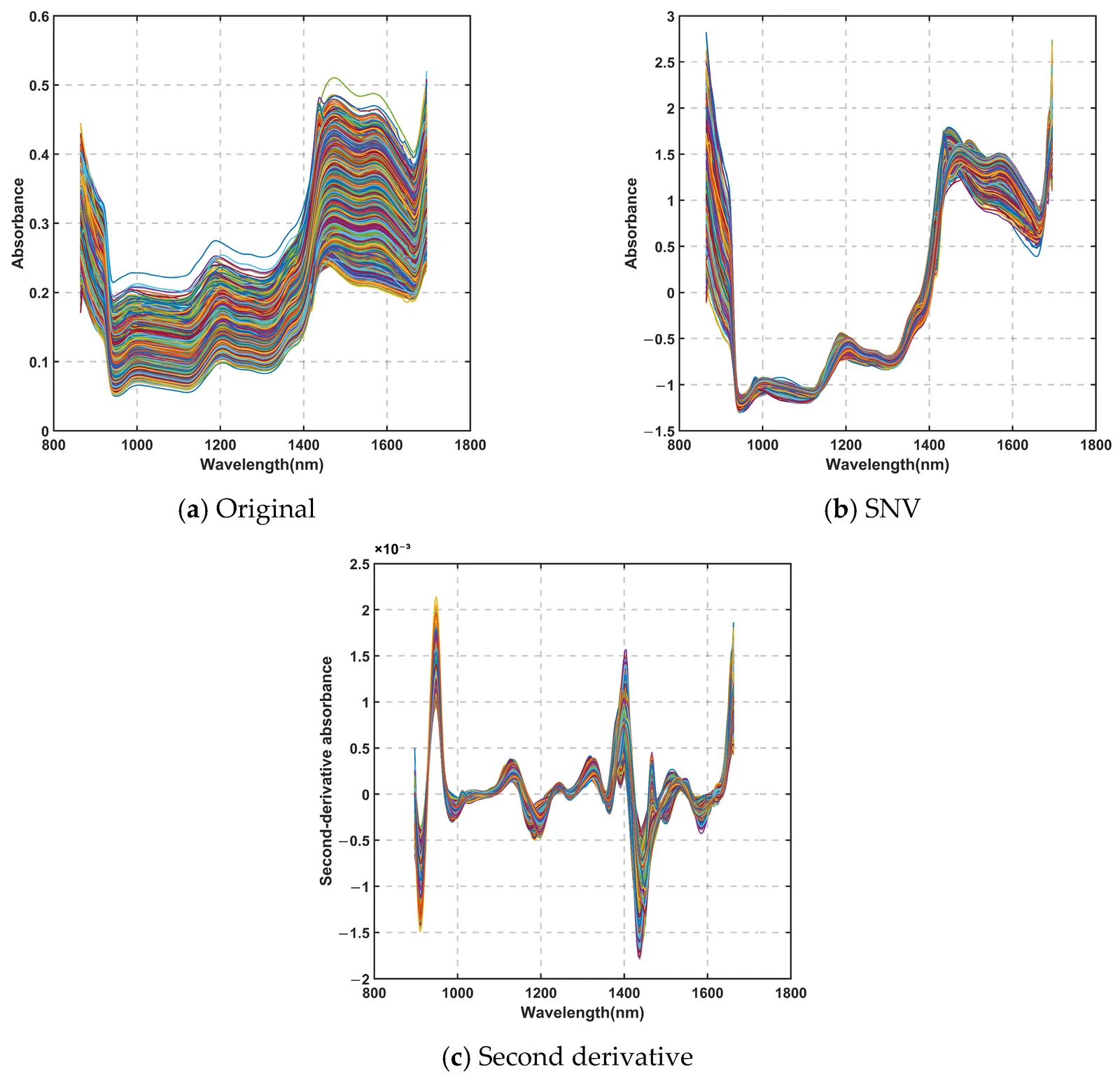
Block-based near-infrared spectra (9 × 9 pixels) obtained from the development samples: (**a**) Original spectra, (**b**) SNV preprocessed spectra, and (**c**) Second-derivative spectra. Line colors are used only to visually distinguish overlapping spectra and do not indicate different sample classes or categories.

The corresponding block-based spectra are presented in Figure 3. Compared with the mean-spectrum representation, the block-based spectra exhibited greater within-scan spectral variability because localized spectral information was retained rather than averaged across the entire ROI. This representation therefore preserved spatial heterogeneity arising from local particle distribution and sample packing. As observed for the mean-spectrum data, SNV reduced scattering-related variation, whereas second-derivative preprocessing enhanced localized spectral features.

Although whole-ROI averaging can reduce random local noise, it also compresses the distribution of localized spectral responses and may attenuate or obscure features arising from spatial heterogeneity in particle arrangement, packing, scattering, or composition [12,18].

Taken together, the mean-spectrum representation provided a compact scan-level spectral profile, whereas the block-based representation retained localized spectral variability and spatial heterogeneity. These contrasting representations were subsequently evaluated to determine their effects on classification performance.

### 3.2. Comparison of Classification Model Performance

Classification performance was first evaluated using LOPSO-CV on the development dataset. In each fold, all four independently sampled and repacked scans originating from the same physical sample, together with all associated blocks, were withheld from model training. The resulting four scan-level prediction scores were then averaged to obtain one physical-sample-level prediction. Accordingly, the internal performance metrics reported in Table 2 were calculated from 90 independent physical-sample predictions.

For the mean-spectrum representation, PLS-DA showed excellent internal performance. Both the original and SNV models achieved perfect classification of the 90 physical samples, whereas the second-derivative model achieved an accuracy and balanced accuracy of 0.9889, with a sensitivity of 0.9778 and specificity of 1.0000. Mean-spectrum RF showed somewhat greater dependence on spectral preprocessing. The original model achieved an accuracy and balanced accuracy of 0.9111, whereas SNV and second-derivative preprocessing both improved accuracy to 0.9778. These results indicate that whole-ROI spectral averaging provided strong discrimination within the development dataset, although performance varied according to classifier and preprocessing condition.

For the block-based representation, block-size sensitivity was evaluated using 7 × 7, 9 × 9, and 11 × 11 pixel blocks under the same LOPSO-CV framework (Table 2). The three configurations yielded 165,747, 96,728, and 62,522 valid blocks, respectively. Overall classification performance was similar for the 9 × 9 and 11 × 11 configurations, whereas the 7 × 7 configuration generally showed slightly lower performance. Across the evaluated classifier and preprocessing combinations, the mean balanced accuracies were approximately 0.9667, 0.9741, and 0.9766 for the 7 × 7, 9 × 9, and 11 × 11 configurations, respectively. Thus, the difference in overall classification performance between the 9 × 9 and 11 × 11 configurations was small, whereas the 9 × 9 configuration retained a larger number of localized spatial observations. Accordingly, the 9 × 9 block size was selected on the basis of the empirical block-size sensitivity analysis rather than being fixed a priori, providing a practical compromise between spectral averaging for noise reduction and preservation of localized spatial information.

Using the selected 9 × 9 representation, block-based PLS-DA achieved accuracies of 0.9444, 0.9222, and 0.9667 for the original, SNV, and second-derivative spectra, respectively. The second-derivative model provided the strongest overall performance, with a sensitivity of 0.9333, specificity of 1.0000, balanced accuracy of 0.9667, AUC of 0.9956, precision of 1.0000, and F1-score of 0.9655. Block-based RF showed consistently high performance, with accuracies of 0.9667, 0.9889, and 0.9778 for the original, SNV, and second-derivative spectra, respectively. The SNV model provided the highest balanced accuracy (0.9889) and an AUC of 0.9985. MI-ACE achieved error-free physical-sample-level classification under all three preprocessing conditions in the development dataset.

Although individual blocks were used as spectral observations during block-based model fitting, these blocks were nested within scans and physical samples and should not be interpreted as independent experimental units. For the selected 9 × 9 representation, each physical sample contributed 1074.76 ± 10.85 valid blocks across its four scans, with a range of 1049–1100 blocks. This narrow distribution indicates that variation in the number of valid blocks among physical samples was limited and therefore reduced the potential for substantial disproportionate contribution arising solely from unequal block counts. Nevertheless, independence for model evaluation was maintained at the physical-sample level through LOPSO-CV rather than at the block level.

Independent external validation was subsequently performed using 21 additional physical samples that were not used during model development. For each physical sample, the continuous prediction scores from its four independently sampled and repacked scans were averaged before class assignment. Thus, the external-validation metrics in Table 3 were calculated from 21 independent physical-sample predictions rather than from the 84 individual scans. Mean-spectrum PLS-DA showed lower external-validation performance than during internal cross-validation, with accuracies of 0.8095, 0.7143, and 0.8571 for the original, SNV, and second-derivative spectra, respectively. In contrast, mean-spectrum RF correctly classified all 21 physical samples under all three preprocessing conditions.

The 9 × 9 block-based models also showed strong external-validation performance. Block-based PLS-DA achieved accuracies of 1.0000, 0.9048, and 1.0000 for the original, SNV, and second-derivative spectra, respectively. Block-based RF and MI-ACE correctly classified all 21 physical samples under all three preprocessing conditions. The corresponding sensitivity, specificity, balanced accuracy, AUC, precision, F1-score, and 95% confidence intervals are reported in Table 3. Although several models produced point estimates of 1.0000, the associated confidence intervals remained dependent on the limited number of independent physical samples. For example, error-free classification of all 21 samples corresponded to an accuracy of 1.0000 with an exact 95% binomial confidence interval of 0.8389–1.0000, emphasizing that perfect classification within this dataset should not be interpreted as zero uncertainty.

The physical-sample-level confusion matrices are summarized in Table 4. The external-validation dataset comprised 15 samples assigned to the EBN-positive class and six EBN-free samples. The selected 9 × 9 block-based PLS-DA with second-derivative preprocessing, 9 × 9 block-based RF with SNV preprocessing, and 9 × 9 block-based MI-ACE using original spectra each yielded TP = 15, TN = 6, FP = 0, and FN = 0. Table 5 further summarizes performance across the external-validation subgroups. All three selected models correctly classified 3/3 laboratory-prepared samples at each nominal EBN level of 0.1, 0.5, and 1.0%, 2/2 samples from each of the three commercial brands, and 3/3 samples from each of the KA and SO control groups. Because each subgroup contained only two or three independent physical samples, these subgroup results should be regarded as descriptive evidence rather than precise estimates of subgroup-specific performance.

The difference between the mean-spectrum and block-based results can be interpreted in terms of how the same hyperspectral data were represented during model fitting. In the mean-spectrum approach, all valid localized spectra within a scan were averaged before classification, providing a compact representation but removing information on the distribution of localized spectral responses. For heterogeneous powder materials, such averaging may obscure localized variation that remains available in spatially resolved spectra [12,18]. In contrast, the block-based approach retained localized spectral observations during model training and subsequently aggregated the resulting continuous prediction scores at the scan and physical-sample levels. Consequently, mean-spectrum and block-based models were fitted to different representations of the same hyperspectral scans and should not be expected to produce equivalent decision boundaries or latent structures [26].

The external-validation results do not, however, indicate that the block-based representation was uniformly superior for all classifiers. The largest difference was observed for PLS-DA, for which the selected 9 × 9 s-derivative block-based model classified all 21 external samples correctly, whereas the corresponding mean-spectrum second-derivative model achieved an accuracy of 0.8571. RF performed strongly with both representations and achieved error-free classification for all mean-spectrum and 9 × 9 block-based preprocessing conditions in the present external-validation set. Thus, the benefit of localized spectral representation appears to depend partly on classifier structure and preprocessing rather than representing a universal improvement over whole-ROI averaging. For RF, access to a broader distribution of localized spectral observations may facilitate modeling of nonlinear spectral relationships [27], whereas MI-ACE explicitly accommodates uncertainty in instance-level labels within its multiple-instance learning formulation [29].

Although several models achieved error-free classification in the external-validation set, these findings should be interpreted as proof-of-concept performance within the evaluated sample set rather than evidence of broad generalizability. The external-validation dataset included only two EBN-free matrices (KA and SO), laboratory-prepared EBN formulations based on material obtained from a single EBN source, and six commercial products representing three brands. Furthermore, the commercial products were assigned to the EBN-positive class according to their label-declared EBN content without independent chemical or molecular confirmation. Consequently, some of the observed discrimination may partly reflect source- or matrix-specific spectral characteristics rather than universally generalizable EBN-specific information. Broader validation using EBN from multiple geographic origins and production batches, additional commercial formulations, and a broader range of realistic adulterants and substitute matrices will therefore be required. In particular, protein-containing materials such as egg white should be included to more rigorously assess the specificity and broader generalizability of the proposed approach.

Because the actual chemical composition of the dried solid fractions was not independently quantified after filtration, washing, and drying, the present study cannot directly relate the chemometric predictions to chemically verified EBN composition in the analyzed powder fractions. Future studies should incorporate orthogonal chemical measurements of the same physical samples to evaluate the relationship between model predictions and independently measured compositional markers.

From a practical perspective, the present workflow should be distinguished from rapid screening of intact ready-to-drink beverages. Although NIR-HSI acquisition itself provides spatially resolved spectral information without requiring chemical reagents during measurement, the complete analytical procedure requires substantial sample preparation. Oven drying alone required approximately 18–20 h, while filtration, six washing cycles, grinding, and sieving involved additional manual handling. The standardized preparation was intended to reduce spectral contributions from water and readily soluble formulation components and to produce a more uniform solid fraction for analysis, which may reduce matrix-related variability and facilitate classification. However, these advantages are accompanied by increased preparation time and labor. Therefore, the present approach is more appropriately considered a laboratory-based authentication procedure for prepared beverage-derived powder fractions than a rapid, direct, or non-invasive screening method for commercial beverages in their original state. Future work should investigate whether selected preparation steps can be simplified, for example by reducing the number of washing cycles, using a more rapid drying procedure, or minimizing grinding and sieving, as well as whether direct or minimally processed liquid analysis can retain adequate classification performance.

For the subsequent spatial multi-model agreement analysis, one final 9 × 9 block-based model from each classifier family was selected exclusively from the development dataset using LOPSO-CV. Balanced accuracy was used as the primary selection criterion and AUC as the secondary criterion; when performance was equivalent, the least transformed spectral representation was retained as a parsimonious tie-breaking criterion. This procedure resulted in the selection of second-derivative PLS-DA, SNV-preprocessed RF, and original-spectrum MI-ACE. The independent external-validation dataset was not used for model or preprocessing selection. These three models were subsequently used for the spatial agreement mapping described in Section 3.3.

### 3.3. Spatial Distribution of Multi-Model Agreement

**Figure 4:**
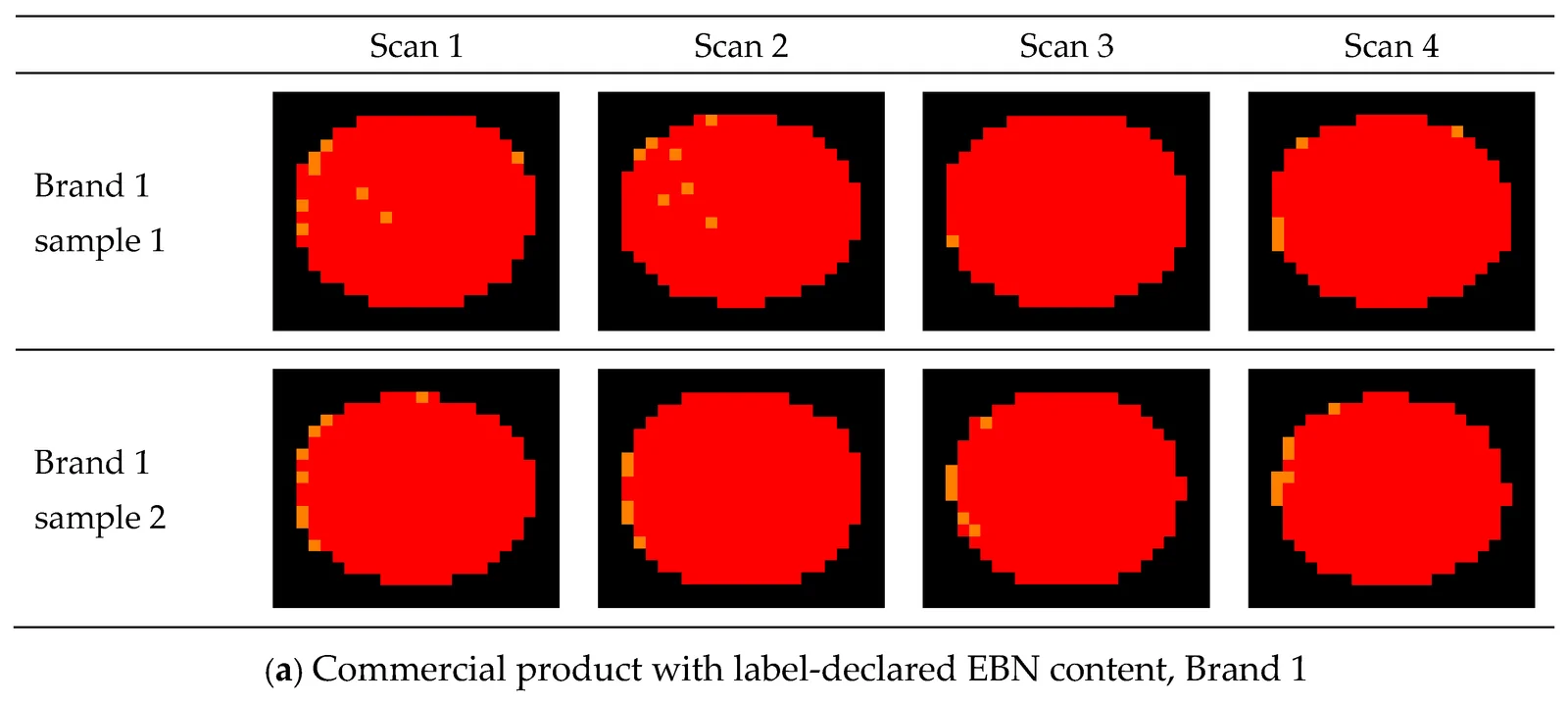

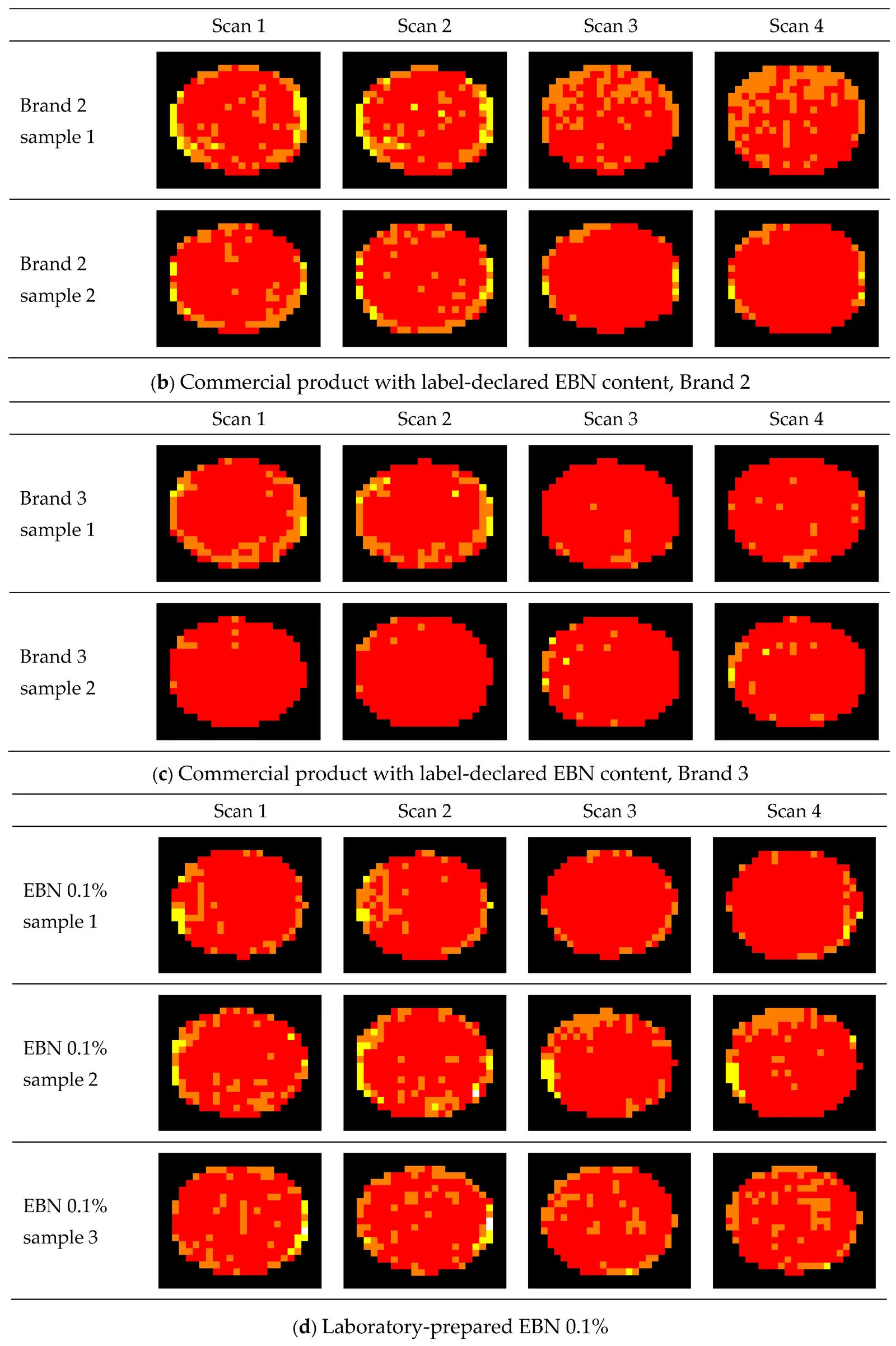

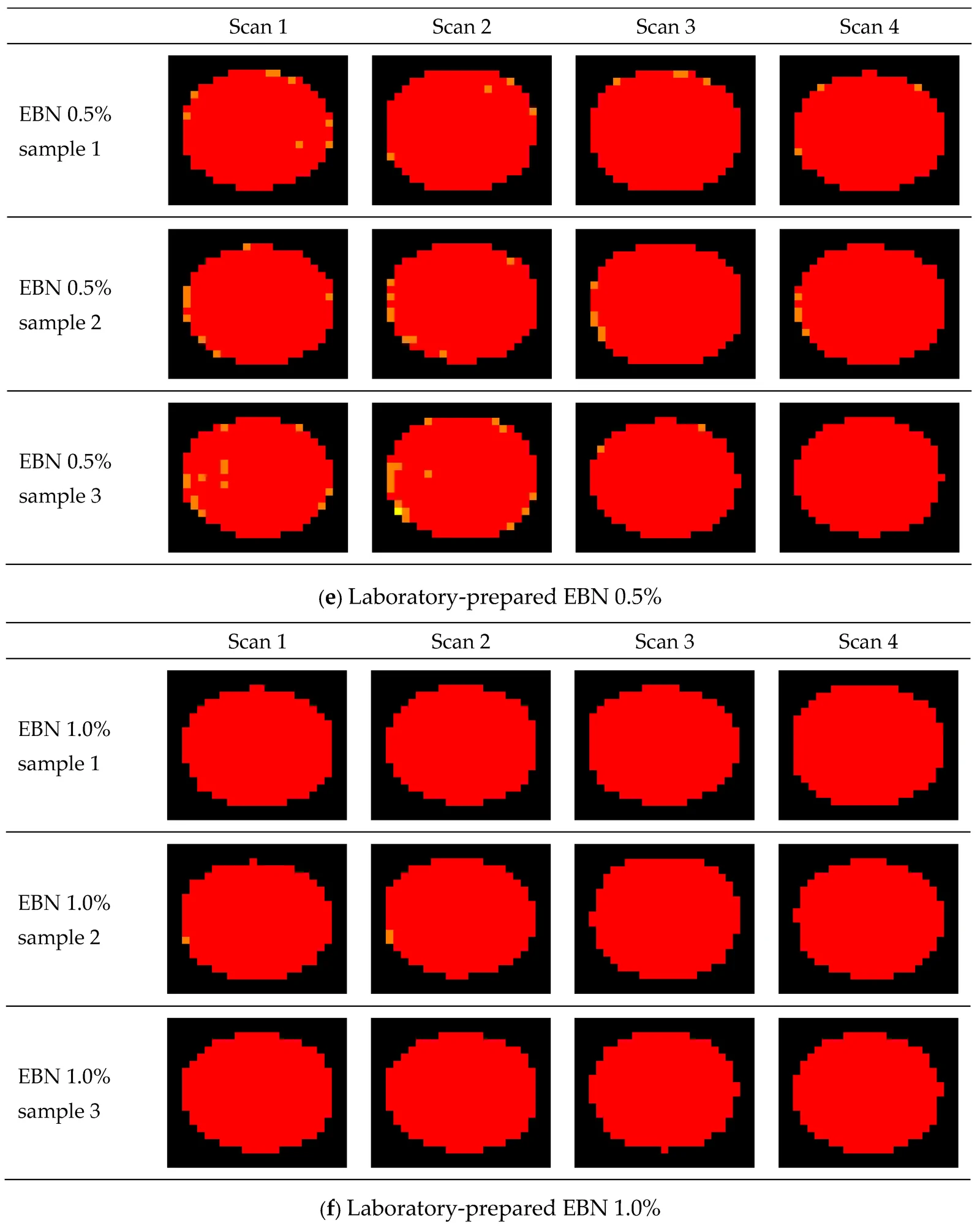

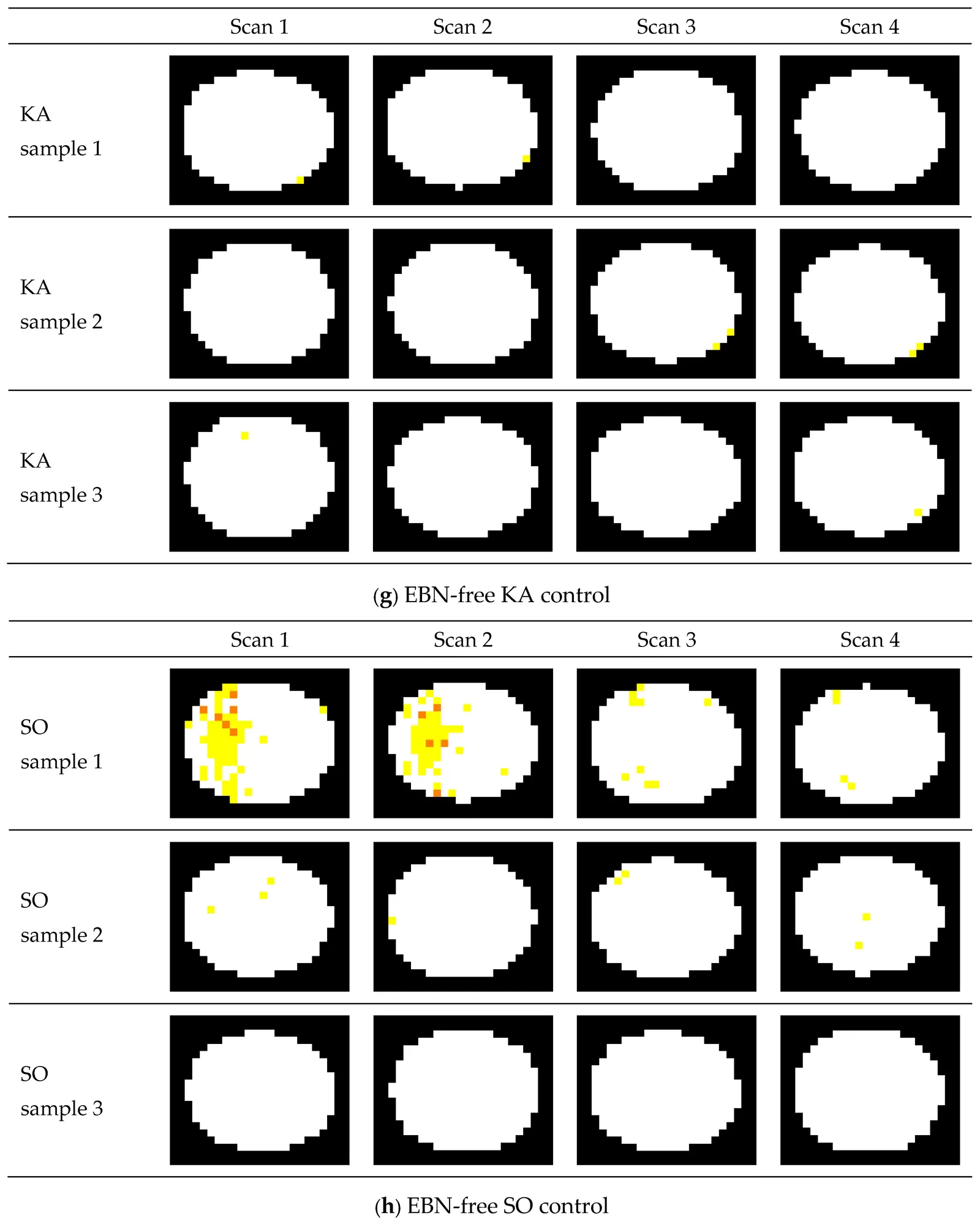
Spatial multi-model agreement maps of EBN-containing and EBN-free beverage-derived powder samples from the independent external validation set. The samples assigned to the EBN-positive class comprised commercial products with label-declared EBN content from (**a**) Brand 1, (**b**) Brand 2, and (**c**) Brand 3, together with laboratory-prepared formulations containing (**d**) 0.1%, (**e**) 0.5%, and (**f**) 1.0% EBN (*w*/*w*). The EBN-free controls consisted of (**g**) KA and (**h**) SO. Two independent physical samples were evaluated for each commercial brand, whereas three independent physical samples were evaluated for each laboratory-prepared EBN level and each EBN-free control group. For each physical sample, four independently sampled aliquots were repacked and scanned separately. Red, orange, yellow, and white indicate agreement scores of 3/3, 2/3, 1/3, and 0/3, respectively, corresponding to the number of models classifying each block as EBN-positive. Black areas represent the background outside the ROI.

The spatial model-agreement maps generated from the selected 9 × 9 block-based second-derivative PLS-DA, SNV-preprocessed RF, and original-spectrum MI-ACE models are shown in Figure 4. The maps revealed spatial variation in the level of agreement among the three classifiers within individual powder samples.

Variations in agreement patterns were observed among the four scans of some physical samples. Because each scan was obtained from a newly sampled powder aliquot that was independently repacked before image acquisition, these differences may reflect local spectral heterogeneity within the powder matrix together with variability associated with subsampling and repacking.

In the EBN-containing samples, a large proportion of the ROI consisted of 3/3 consensus-positive regions, indicating that all three models classified the corresponding blocks as EBN-positive. Some samples also contained 2/3 and 1/3 agreement regions, demonstrating localized differences in classifier responses within the samples.

The EBN-free KA and SO controls were dominated by 0/3 consensus-negative regions, consistent with their known sample-level class. A small number of isolated 1/3 regions were also observed, indicating localized disagreement among the classifiers. These isolated block-level differences did not affect the final physical-sample-level classification because block-level scores were first averaged within each scan, after which the four scan-level scores from the same physical sample were averaged before final class assignment. To quantitatively characterize the similarity and diversity among the three selected classifiers, pairwise block-level agreement, disagreement, and Cohen’s kappa were calculated across the 22,706 valid blocks from the independent external-validation set (Table 6).

Pairwise block-level analysis showed that PLS-DA and RF achieved 94.0412% agreement and 5.9588% disagreement (Cohen’s kappa = 0.86224), while PLS-DA and MI-ACE showed 95.0718% agreement and 4.9282% disagreement (Cohen’s kappa = 0.88434). The highest agreement was observed between RF and MI-ACE, with 97.0757% agreement, 2.9243% disagreement, and a Cohen’s kappa of 0.92943. Thus, the three classifiers produced highly concordant but not identical block-level decisions. These results indicate that the agreement maps capture localized variation among classifier responses; however, the generally high pairwise agreement also indicates that the model outputs are correlated rather than statistically independent. Accordingly, 3/3 regions are interpreted as regions of complete inter-model consensus rather than as regions of independently confirmed prediction confidence. This distinction is also consistent with the broader emphasis on interpretability in modern classification, in which model outputs should be interpreted in relation to the information available to the models rather than treated as inherently independent evidence [32].

To further examine the spectral characteristics associated with these spatial classification patterns, 3/3 consensus-positive regions from EBN-containing samples and 0/3 consensus-negative regions from the KA and SO controls were selected for subsequent spectral characterization.

### 3.4. Spectral Characteristics and Model-Independent ROI Analysis

To further characterize the spectral patterns associated with the spatial model-agreement maps, spectra were initially extracted from regions showing complete inter-model consensus. For EBN-containing samples, blocks classified as EBN-positive by all three selected models (3/3 consensus-positive regions) were included, whereas blocks classified as EBN-negative by all three models (0/3 consensus-negative regions) were selected from the EBN-free KA and SO controls.

Because these regions were defined according to the predictions of the same classifiers used for agreement mapping, the resulting spectral comparison may be influenced by a model-selection effect. Therefore, the consensus-region spectra were treated as descriptive rather than as independent validation of the spectral features associated with EBN-containing samples. To examine whether the observed spectral tendency persisted without model-based region selection, an additional analysis was performed using all valid blocks within the ROI of each independent external-validation sample, irrespective of classifier prediction or agreement score. The all-ROI spectra were averaged at the physical-sample level and subsequently summarized within each sample group.

One sample of 100% ground EBN powder was additionally included as a qualitative spectral reference. This reference was used only for spectral interpretation and was not included in classification model development. Because the EBN-containing samples were beverage-derived powders containing other formulation components, their spectral profiles were not expected to fully correspond to that of the 100% EBN reference.

**Figure 5:**
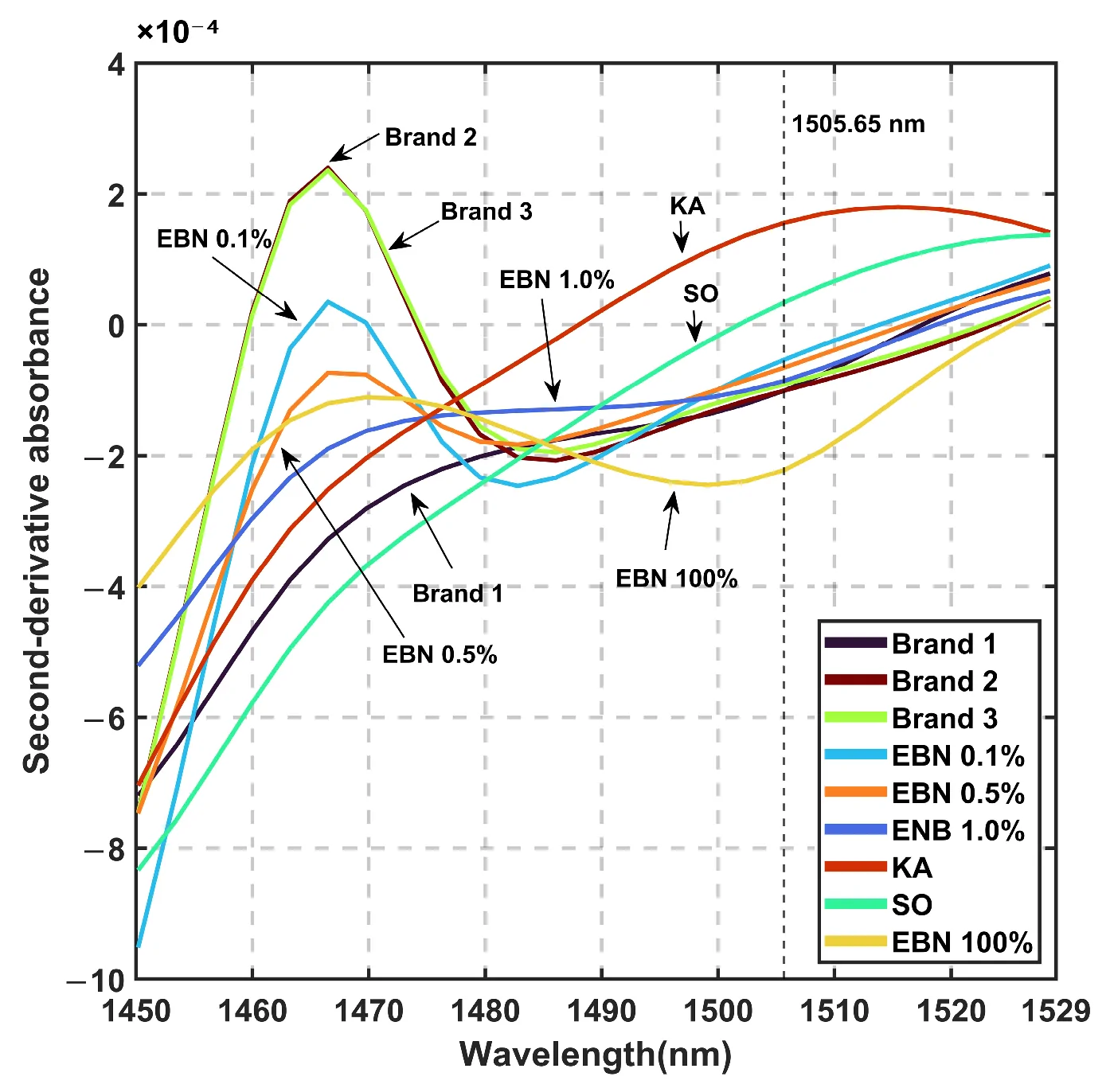
Savitzky–Golay second-derivative spectra in the wavelength range of 1450–1520 nm obtained from all valid ROI blocks of the independent external-validation samples without model-based region selection. Spectra were first averaged at the physical-sample level and subsequently averaged within each sample group. The spectrum of one 100% ground EBN sample was included as a qualitative spectral reference. The dashed line indicates the spectral position at approximately 1505.65 nm used for comparison among sample groups.

Figure 5 shows the Savitzky–Golay second-derivative spectra in the 1450–1520 nm region obtained from all valid ROI blocks of the independent external-validation samples without model-based region selection. At approximately 1505.65 nm, the commercial products with label-declared EBN content and the laboratory-prepared 0.1%, 0.5%, and 1.0% EBN groups exhibited negative second-derivative responses, whereas the EBN-free KA and SO controls exhibited positive responses. Importantly, this directional separation remained evident when all valid ROI blocks were included, indicating that the observed spectral tendency was not restricted to regions selected according to model consensus.

The persistence of this tendency was further evaluated at the physical-sample level using the all-ROI spectra. At 1505.65 nm, all 15 EBN-positive physical samples showed negative second-derivative values, whereas all six EBN-free physical samples showed positive values. The median second-derivative response was −8.87 × 10^−5^ for the EBN-positive group (*n* = 15) and 9.44 × 10^−5^ for the EBN-free group (*n* = 6). An exact two-sided Mann–Whitney U test showed a significant difference between the two groups (U = 0, *p* < 0.001). The same directional separation was retained when the analysis was restricted to the laboratory-prepared EBN samples (*n* = 9) and the EBN-free controls (*n* = 6; U = 0, *p* < 0.001), indicating that the observed difference was not dependent solely on the label-declared commercial samples.

The spectral feature around 1505–1509 nm may be associated with the first overtone of N–H stretching vibrations, which has been reported in protein-containing materials in the approximately 1500–1510 nm region [33,34,35]. However, near-infrared (NIR) absorption bands are broad and overlapping, and the observed response may also be influenced by matrix composition and spectral preprocessing. Moreover, the wavelength region was identified retrospectively from the present dataset, and no orthogonal chemical measurement was available to verify its molecular origin. Accordingly, the 1505–1509 nm region should be regarded as a potentially informative spectral region associated with differences between the evaluated EBN-containing and EBN-free samples rather than as an EBN-specific chemical marker.

## 4. Conclusions

This study developed a block-based NIR-HSI framework for authenticating EBN beverage-derived powders by integrating localized spectral representation, complementary classification models, and spatial multi-model agreement mapping. Mean-spectrum PLS-DA showed high performance during internal cross-validation but lower accuracy during independent external validation, whereas mean-spectrum RF demonstrated stronger external performance.

For the block-based representation, second-derivative PLS-DA achieved an external-validation accuracy of 1.0000, while block-based RF and MI-ACE correctly classified all 21 physical samples under all three preprocessing conditions. These findings suggest that retaining localized spectral information can support strong classification performance for the EBN-containing formulations and EBN-free KA/SO matrices evaluated in this study. However, error-free classification within the present dataset should not be interpreted as evidence of universally perfect or EBN-specific authentication performance.

The development-data-selected block-based models, namely second-derivative PLS-DA, SNV-preprocessed RF, and original-spectrum MI-ACE, were further integrated into a spatial multi-model agreement framework, enabling visualization of localized agreement and disagreement among classifiers. Exploratory spectral analysis identified a potentially informative region around 1505–1509 nm, with a significant difference in second-derivative response at approximately 1505.65 nm between physical samples assigned to the EBN-positive and EBN-free classes. Because no orthogonal chemical confirmation was available, this region should not be interpreted as an EBN-specific chemical marker.

The findings should be regarded as proof-of-concept evidence within the evaluated sample set. External validation was limited to two EBN-free control matrices, laboratory-prepared EBN formulations based on material from a single source, and six commercial products from three brands whose EBN-positive status was based on label declarations rather than independent chemical confirmation. Broader validation using EBN from multiple geographic origins and production batches, additional commercial formulations and substitute matrices, and orthogonal chemical or molecular verification is therefore required before generalizability can be established.

The current workflow also requires substantial sample preparation, including filtration, repeated washing, 18–20 h of oven drying, grinding, and sieving. It should therefore be regarded as a laboratory-based authentication workflow for prepared beverage-derived powders rather than a rapid or non-invasive screening method for intact beverages. Future studies should evaluate simplified sample preparation and, as larger, more diverse, and chemically verified datasets become available, investigate advanced spectral-learning approaches, including multiscale deep ensemble learning [36], diffusion-based spectral modeling [37], and multi-task neural architecture optimization [38]. Overall, the proposed framework provides a basis for combining EBN authentication with spatially resolved interpretation while maintaining evaluation at the independent physical-sample level.

## Figures and Tables

**Table 1:** Composition of laboratory-prepared samples per 500 g batch.

Formulation	EBN (g)	Sodium Alginate (g)	Karaya Gum (g)	Rock Sugar (g)	Gellan Gum (g)	Sucralose (g)	Water (g)	Total (g)
EBN 0.1%	0.50	199.50	–	30.00	0.10	0.03	269.87	500
EBN 0.5%	2.50	197.50	–	30.00	0.10	0.03	269.87	500
EBN 1.0%	5.00	195.00	–	30.00	0.10	0.03	269.87	500
EBN 2.0%	10.00	190.00	–	30.00	0.10	0.03	269.87	500
EBN 3.0%	15.00	185.00	–	30.00	0.10	0.03	269.87	500
SO	–	200.00	–	30.00	0.10	0.03	269.87	500
KA	–	–	200.00	30.00	0.10	0.03	269.87	500

**Table 2:** Physical-sample-level classification performance and selected model parameters obtained from leave-one-physical-sample-out cross-validation of the development dataset, including block-size sensitivity analysis.

Data Representation/Model	Preprocessing	Model Complexity/Parameter	Accuracy	Sensitivity	Specificity	Balanced Accuracy	AUC	F1-Score	Precision
Mean-spectrum PLS-DA	Original	LVs = 13	1.0000	1.0000	1.0000	1.0000	1.0000	1.0000	1.0000
	SNV	LVs = 12	1.0000	1.0000	1.0000	1.0000	1.0000	1.0000	1.0000
	Second derivative	LVs = 10	0.9889	0.9778	1.0000	0.9889	1.0000	0.9888	1.0000
Mean-spectrum RF	Original	Trees = 50; minimum leaf size = 5; predictors/split = 16	0.9111	0.9333	0.8889	0.9111	0.9467	0.9130	0.8936
	SNV	Trees = 50; minimum leaf size = 5; predictors/split = 16	0.9778	0.9556	1.0000	0.9778	1.0000	0.9773	1.0000
	Second derivative	Trees = 50; minimum leaf size = 5; predictors/split = 15	0.9778	0.9556	1.0000	0.9778	0.9980	0.9773	1.0000
7 × 7 Block-based PLS-DA	Original	LVs = 11	0.9444	0.9333	0.9556	0.9444	0.9891	0.9438	0.9545
	SNV	LVs = 7	0.9222	0.9111	0.9333	0.9222	0.9857	0.9213	0.9318
	Second derivative	LVs = 7	0.9333	0.9111	0.9556	0.9333	0.9867	0.9318	0.9535
9 × 9 Block-based PLS-DA	Original	LVs = 12	0.9444	0.9333	0.9556	0.9444	0.9916	0.9438	0.9545
	SNV	LVs = 11	0.9222	0.8889	0.9556	0.9222	0.9916	0.9195	0.9524
	Second derivative	LVs = 10	0.9667	0.9333	1.0000	0.9667	0.9956	0.9655	1.0000
11 × 11 Block-based PLS-DA	Original	LVs = 14	0.9556	0.9556	0.9556	0.9556	0.9970	0.9556	0.9556
	SNV	LVs = 12	0.9222	0.8889	0.9556	0.9222	0.9936	0.9195	0.9524
	Second derivative	LVs = 10	0.9667	0.9333	1.0000	0.9667	0.9970	0.9655	1.0000
7 × 7 Block-based RF	Original	Trees = 100; minimum leaf size = 5; predictors/split = 16	0.9444	0.9778	0.9111	0.9444	0.9931	0.9462	0.9167
	SNV	Trees = 50; minimum leaf size = 5; predictors/split = 16	0.9778	0.9556	1.0000	0.9778	0.9985	0.9773	1.0000
	Second derivative	Trees = 50; minimum leaf size = 1; predictors/split = 15	0.9778	0.9778	0.9778	0.9778	0.9975	0.9778	0.9778
9 × 9 Block-based RF	Original	Trees = 100; minimum leaf size = 5; predictors/split = 32	0.9667	0.9778	0.9556	0.9667	0.9911	0.9670	0.9565
	SNV	Trees = 100; minimum leaf size = 5; predictors/split = 32	0.9889	0.9778	1.0000	0.9889	0.9985	0.9888	1.0000
	Second derivative	Trees = 50; minimum leaf size = 5; predictors/split = 31	0.9778	0.9778	0.9778	0.9778	0.9985	0.9778	0.9778
11 × 11 Block-based RF	Original	Trees = 50; minimum leaf size = 5; predictors/split = 31	0.9667	0.9778	0.9556	0.9667	0.9951	0.9670	0.9565
	SNV	Trees = 100; minimum leaf size = 5; predictors/split = 16	0.9889	0.9778	1.0000	0.9889	0.9995	0.9888	1.0000
	Second derivative	Trees = 100; minimum leaf size = 5; predictors/split = 15	0.9889	0.9778	1.0000	0.9889	0.9990	0.9888	1.0000
7 × 7 Block-based MI-ACE	Original	λ = 10^−4^; max training blocks/scan = 25; threshold = 0.212494	1.0000	1.0000	1.0000	1.0000	1.0000	1.0000	1.0000
	SNV	λ = 10^−4^; max training blocks/scan = 25; threshold = 0.199755	1.0000	1.0000	1.0000	1.0000	1.0000	1.0000	1.0000
	Second derivative	λ = 10^−2^; max training blocks/scan = 25; threshold = 0.239875	1.0000	1.0000	1.0000	1.0000	1.0000	1.0000	1.0000
9 × 9 Block-based MI-ACE	Original	λ = 10^−4^; max training blocks/scan = 25; threshold = 0.230752	1.0000	1.0000	1.0000	1.0000	1.0000	1.0000	1.0000
	SNV	λ = 10^−4^; max training blocks/scan = 25; threshold = 0.218887	1.0000	1.0000	1.0000	1.0000	1.0000	1.0000	1.0000
	Second derivative	λ = 10^−2^; max training blocks/scan = 25; threshold = 0.247352	1.0000	1.0000	1.0000	1.0000	1.0000	1.0000	1.0000
11 × 11 Block-based MI-ACE	Original	λ = 10^−4^; max training blocks/scan = 25; threshold = 0.235159	1.0000	1.0000	1.0000	1.0000	1.0000	1.0000	1.0000
	SNV	λ = 10^−2^; max training blocks/scan = 25; threshold = 0.236069	1.0000	1.0000	1.0000	1.0000	1.0000	1.0000	1.0000
	Second derivative	λ = 10^−2^; max training blocks/scan = 25; threshold = 0.256215	1.0000	1.0000	1.0000	1.0000	1.0000	1.0000	1.0000

Block-size sensitivity was evaluated using 7 × 7, 9 × 9, and 11 × 11 spatial blocks under LOPSO-CV. Across the evaluated model and preprocessing combinations, overall classification performance was comparable between the 9 × 9 and 11 × 11 configurations, whereas the 7 × 7 blocks generally showed slightly lower performance. The 9 × 9 configuration was therefore retained as a compromise between spectral averaging for noise reduction and preservation of localized spatial information, rather than because it produced uniformly superior performance across all models. In the development dataset, the 7 × 7, 9 × 9, and 11 × 11 configurations yielded 165,747, 96,728, and 62,522 valid blocks, respectively. For the selected 9 × 9 configuration, 268.69 ± 3.52 valid blocks were retained per scan (range: 259–277).

**Table 3:** Physical-sample-level classification performance with 95% confidence intervals for the independent external-validation set comprising 21 physical samples.

Model	Preprocessing	Accuracy (95% CI)	Sensitivity (95% CI)	Specificity (95% CI)	Balanced Accuracy (95% CI)	AUC (95% CI)	F1-Score (95% CI)	Precision (95% CI)
Mean-spectrum PLS-DA	Original	0.8095 (0.5809–0.9455)	0.7333 (0.4490–0.9221)	1.0000 (0.5407–1.0000)	0.8667 (0.7333–0.9667)	0.9444 (0.8222–1.0000)	0.8462 (0.6364–0.9655)	1.0000 (0.7151–1.0000)
	SNV	0.7143 (0.4782–0.8872)	0.6000 (0.3229–0.8366)	1.0000 (0.5407–1.0000)	0.8000 (0.6667–0.9333)	0.8889 (0.7222–1.0000)	0.7500 (0.5000–0.9286)	1.0000 (0.6637–1.0000)
	Second derivative	0.8571 (0.6366–0.9695)	0.8000 (0.5191–0.9567)	1.0000 (0.5407–1.0000)	0.9000 (0.8000–1.0000)	1.0000 (1.0000–1.0000)	0.8889 (0.7500–1.0000)	1.0000 (0.7354–1.0000)
Mean-spectrum RF	Original	1.0000 (0.8389–1.0000)	1.0000 (0.7820–1.0000)	1.0000 (0.5407–1.0000)	1.0000 (1.0000–1.0000)	1.0000 (1.0000–1.0000)	1.0000 (1.0000–1.0000)	1.0000 (0.7820–1.0000)
	SNV	1.0000 (0.8389–1.0000)	1.0000 (0.7820–1.0000)	1.0000 (0.5407–1.0000)	1.0000 (1.0000–1.0000)	1.0000 (1.0000–1.0000)	1.0000 (1.0000–1.0000)	1.0000 (0.7820–1.0000)
	Second derivative	1.0000 (0.8389–1.0000)	1.0000 (0.7820–1.0000)	1.0000 (0.5407–1.0000)	1.0000 (1.0000–1.0000)	1.0000 (1.0000–1.0000)	1.0000 (1.0000–1.0000)	1.0000 (0.7820–1.0000)
9 × 9 Block-based PLS-DA	Original	1.0000 (0.8389–1.0000)	1.0000 (0.7820–1.0000)	1.0000 (0.5407–1.0000)	1.0000 (1.0000–1.0000)	1.0000 (1.0000–1.0000)	1.0000 (1.0000–1.0000)	1.0000 (0.7820–1.0000)
	SNV	0.9048 (0.6962–0.9883)	0.8667 (0.5954–0.9834)	1.0000 (0.5407–1.0000)	0.9333 (0.8333–1.0000)	1.0000 (1.0000–1.0000)	0.9286 (0.8000–1.0000)	1.0000 (0.7529–1.0000)
	Second derivative	1.0000 (0.8389–1.0000)	1.0000 (0.7820–1.0000)	1.0000 (0.5407–1.0000)	1.0000 (1.0000–1.0000)	1.0000 (1.0000–1.0000)	1.0000 (1.0000–1.0000)	1.0000 (0.7820–1.0000)
9 × 9 Block-based RF	Original	1.0000 (0.8389–1.0000)	1.0000 (0.7820–1.0000)	1.0000 (0.5407–1.0000)	1.0000 (1.0000–1.0000)	1.0000 (1.0000–1.0000)	1.0000 (1.0000–1.0000)	1.0000 (0.7820–1.0000)
	SNV	1.0000 (0.8389–1.0000)	1.0000 (0.7820–1.0000)	1.0000 (0.5407–1.0000)	1.0000 (1.0000–1.0000)	1.0000 (1.0000–1.0000)	1.0000 (1.0000–1.0000)	1.0000 (0.7820–1.0000)
	Second derivative	1.0000 (0.8389–1.0000)	1.0000 (0.7820–1.0000)	1.0000 (0.5407–1.0000)	1.0000 (1.0000–1.0000)	1.0000 (1.0000–1.0000)	1.0000 (1.0000–1.0000)	1.0000 (0.7820–1.0000)
9 × 9 Block-based MI-ACE	Original	1.0000 (0.8389–1.0000)	1.0000 (0.7820–1.0000)	1.0000 (0.5407–1.0000)	1.0000 (1.0000–1.0000)	1.0000 (1.0000–1.0000)	1.0000 (1.0000–1.0000)	1.0000 (0.7820–1.0000)
	SNV	1.0000 (0.8389–1.0000)	1.0000 (0.7820–1.0000)	1.0000 (0.5407–1.0000)	1.0000 (1.0000–1.0000)	1.0000 (1.0000–1.0000)	1.0000 (1.0000–1.0000)	1.0000 (0.7820–1.0000)
	Second derivative	1.0000 (0.8389–1.0000)	1.0000 (0.7820–1.0000)	1.0000 (0.5407–1.0000)	1.0000 (1.0000–1.0000)	1.0000 (1.0000–1.0000)	1.0000 (1.0000–1.0000)	1.0000 (0.7820–1.0000)

Values are reported at the physical-sample level (*n* = 21). Exact 95% binomial confidence intervals (Clopper–Pearson) were calculated for accuracy, sensitivity, specificity, and precision. Confidence intervals for balanced accuracy, AUC, and F1-score were estimated using 10,000 stratified bootstrap resamples at the physical-sample level. For error-free classifications or perfectly ranked scores, bootstrap intervals for some composite metrics may remain degenerate at 1.0000–1.0000; these intervals should not be interpreted as indicating zero population-level uncertainty.

**Table 4:** Physical-sample-level confusion-matrix results for the independent external-validation set comprising 21 physical samples.

Model	Preprocessing	TP	TN	FP	FN
Mean-spectrum PLS-DA	Original	11	6	0	4
	SNV	9	6	0	6
	Second derivative	12	6	0	3
Mean-spectrum RF	Original	15	6	0	0
	SNV	15	6	0	0
	Second derivative	15	6	0	0
9 × 9 Block-based PLS-DA	Original	15	6	0	0
	SNV	13	6	0	2
	Second derivative	15	6	0	0
9 × 9 Block-based RF	Original	15	6	0	0
	SNV	15	6	0	0
	Second derivative	15	6	0	0
9 × 9 Block-based MI-ACE	Original	15	6	0	0
	SNV	15	6	0	0
	Second derivative	15	6	0	0

TP, true positive; TN, true negative; FP, false positive; FN, false negative. The external-validation set comprised 15 samples assigned to the EBN-positive class and 6 EBN-free samples. Classification was performed at the physical-sample level after averaging the continuous prediction scores from the four independently sampled and repacked scans of each physical sample.

**Table 5:** Physical-sample-level classification results of the selected final block-based models across external-validation subgroups.

External Subgroup	No. of Physical Samples (*n*)	9 × 9 PLS-DA (Second Derivative)	9 × 9 RF (SNV)	9 × 9 MI-ACE (Original)
Laboratory EBN 0.1%	3	3/3	3/3	3/3
Laboratory EBN 0.5%	3	3/3	3/3	3/3
Laboratory EBN 1.0%	3	3/3	3/3	3/3
Commercial Brand 1	2	2/2	2/2	2/2
Commercial Brand 2	2	2/2	2/2	2/2
Commercial Brand 3	2	2/2	2/2	2/2
KA control	3	3/3	3/3	3/3
SO control	3	3/3	3/3	3/3

Values indicate the number of correctly classified physical samples relative to the total number of physical samples in each subgroup. Predictions were obtained after averaging the continuous prediction scores from the four independently sampled and repacked scans of each physical sample. Commercial samples were assigned to the EBN-positive class based on label-declared EBN content and were not independently confirmed by chemical analysis. Owing to the small subgroup sizes (*n* = 2–3), these results are descriptive and should not be interpreted as precise subgroup-specific performance estimates.

**Table 6:** Pairwise block-level agreement and disagreement among the selected models in the independent external-validation set.

Model Pair	No. of Blocks	Agreement (%)	Disagreement (%)	Cohen’s Kappa	Both Negative (0/0)	A−, B+ (0/1)	A+, B− (1/0)	Both Positive (1/1)
9 × 9 PLS-DA (Second derivative) vs. 9 × 9 RF (SNV)	22,706	94.0412	5.9588	0.86224	6495	983	370	14,858
9 × 9 PLS-DA (Second derivative) vs. 9 × 9 MI-ACE (Original)	22,706	95.0718	4.9282	0.88434	6398	1080	39	15,189
9 × 9 RF (SNV) vs. 9 × 9 MI-ACE (Original)	22,706	97.0757	2.9243	0.92943	6319	546	118	15,723

The 22,706 valid blocks were obtained from 84 independently sampled and repacked scans belonging to 21 external-validation physical samples. Pairwise block-level agreement statistics are presented descriptively to characterize model diversity and should not be interpreted as performance estimates based on 22,706 independent experimental samples, because blocks originating from the same physical sample are not statistically independent.

## Data Availability

The processed spectral datasets, physical-sample and scan identifiers, external-validation predictions, and analysis code supporting the findings of this study are not publicly deposited because the research materials and associated data are subject to institutional and funding-agency ownership and data-governance requirements. These materials may be made available from the corresponding author upon reasonable request, subject to applicable institutional and funding-agency approval.

## Abbreviations

The following abbreviations are used in this manuscript:

ACE	Adaptive cosine estimator
AUC	Area under the receiver operating characteristic curve
CI	Confidence interval
DNA	Deoxyribonucleic acid
EBN	Edible bird’s nest
FP	False positive
FN	False negative
Inf	Infinity
KA	Karaya gum
KAPI	Kasetsart Agricultural and Agro-Industrial Product Improvement Institute
KURDI	Kasetsart University Research and Development Institute
LOPSO-CV	Leave-one-physical-sample-out cross-validation
LVs	Latent variables
MI-ACE	Multiple Instance Adaptive Cosine Estimator
NaN	Not a Number
NIR	Near-infrared
NIR-HSI	Near-infrared hyperspectral imaging
PLS-DA	Partial least squares discriminant analysis
RF	Random forest
ROI	Region of interest
SNR	Signal-to-noise ratio
SNV	Standard normal variate
SO	Sodium alginate
TN	True negative
TP	True positive
